# Repurposing Individualized Nutritional Intervention as a Therapeutic Component to Prevent the Adverse Effects of Radiotherapy in Patients With Cervical Cancer

**DOI:** 10.3389/fonc.2020.595351

**Published:** 2020-12-08

**Authors:** Ana Karen Medina-Jiménez, Rebeca Monroy-Torres

**Affiliations:** ^1^ Laboratory of Environmental Nutrition and Food Safety, Medicine and Nutrition Department, University of Guanajuato, Guanajuato, Mexico; ^2^ Observatorio Universitario de Seguridad Alimentaria y Nutricional del Estado de Guanajuato, Guanajuato, Mexico

**Keywords:** ****nutritional intervention, cervical cancer, body composition, radiotherapy, drug repurposing

## Abstract

Worldwide, cervical cancer was the fourth leading cause of cancer death among women, while in Mexico was the second cause (5.28%). Cancer patients receiving chemotherapy and radiotherapy have a high risk of malnutrition secondary to the disease and treatment, affects the patient’s overall, with adverse effects on gastrointestinal symptoms. These use affects the medical therapy. The aim of the present study was to evaluate the benefits on individualized nutritional therapy on decrease weight loss and gastrointestinal adverse effects and to consider these outcomes in pharmacology research, especially in repurposing drugs. We conducted a longitudinal design with two comparation groups with medical diagnosis of cervical cancer and received radiotherapy weekly, 1) the intervention group (nutritional intervention and counseling -INC-) with 20 participants and 2) control group (retrospective cohort -CG-) with 9 participants. Weekly body composition, dietary intake, adverse effects (gastrointestinal symptoms), glucose, hemoglobin, and blood pressure were analyzed during 4 to 5 weeks. Both groups had weight loss weekly (p = 0.013 and p = 0.043 respectively) but the CG vs INC presented loss fat-free mass ≥500g in 67 and of 37% respectively. By the end of the intervention a 25% of the INC group had <10 g/dL of hemoglobin vs 60% for the CG. To compare the dietary intake of vitamins (A and folic acid), fiber (p = 0.006), iron (p = 0.03) and energy (mainly carbohydrates) (p = 0.04) were according to the recommendations in INC group (p>0.05). The number needed to treat was 4 (95% CI, 2 to 13). The nutritional intervention and counseling weekly during radiotherapy in cervical cancer to maintain/improve muscle mass, hemoglobin, and dietary intake above 70% of the recommendations for INC group compared to the evidence. Adequate nutritional status was maintained and decrease the rate of complications, mainly gastrointestinal symptoms, in INC group. The efficacy of drug repurposing can improve through individualized nutritional therapy for preventing adverse effects of radiotherapy in patients with cervical cancer.

## Introduction

Cervical cancer (CC)was the fourth leading cause of cancer death (311,000 deaths) among women in worldwide while in Mexico was the second cause (5.28%) in 2018. The mortality was three times higher in Latin America and the Caribbean compared to North America (1). Women with ages 25 to 69 years and in lower socio-economic groups are more prevalent (2). Some health programs benefit the prevention of chronic degenerative diseases whose main risk factor is overweight and obesity (3). In Mexico vaginal cytology and human papiloma virus (HPV) vaccination are part of CC prevention as part of early detection programs. HPV is the main risk factor (96.6%) of CC (4, 5). There are more than 100 variants of HPV but only the 16 and 18 are associated to CC (70 to 76%) (6).The risk of having HPV increases from 2 to 10 times with the onset of sexual activity (It is exacerbated with greater number of sexual partners), an onset of sexual life before 18 years, adolescent pregnancy, multiparity and smoking (7, 8).

Nutritional intervention and individualized counseling (INC) are a nutritional therapy with dietary prescription based on the control of symptoms for avoiding the undernutrition, overnutrition or any deterioration of the patient. Unfortunately, there is not enough evidence on long term compliance and long term follow up. An INC has benefits in the treatment of many diseases and in this case, it will depend on the type of cancer or its stage (9). There is evidence the INC well implemented impacts and contributes to improve the prognosis of cancer treatment (chemotherapy and radiotherapy) but there is little evidence in cervical cancer (10).

It is known that healthy dietary habits can contribute to reduce CC risk trough maintenance immune system response due to antioxidant presence, avoiding susceptibility to infectious diseases such as HPV (11). The western diet (ultraprocessed foods and sugary drinks, low in fiber, high in saturated fat, sodium, additives) increases the risk of CC (OR = 3.26, 95% CI = 1.03, 10.3; p <0.05) (12). Some studies had associated deficiency of acid folic also other nutrients with a lack immune response (OR = 14.9, 95% CI = 2.65–84.38 and OR = 8.72, 95% CI = 1.55–48.82) (13), vitamin B12 (OR = 0.25, 95% CI, 0.10–0.58, p <0.01 and OR = 0.40, 95% CI, 0.17–0.88, p = 0.02) with an increased risk in prevalence of CC (14). The vitamin C intake has been associated with a decreased risk of cervical intraepithelial neoplasms (OR = 0.58, 95% CI = 0.38–0.89, p = 0.011; OR = 0.59, 95% CI: 0.39–0.89; OR = 0.59, 95% CI = 0.39–0.88 and OR = 0.62 95% CI = 0.40–0.95) (15), as well as the consumption of vegetables and fruits (OR = 0.50, 95% CI = 0.27–0.95). An inverse association between serum levels of carotenoids and tocopherol has high risk of cervical neoplasia (OR = 0.71, 95% CI = 0.56–0.92; p = 0.003 and OR = 0.75, 95% CI = 0.60–0.94; p = 0.008) (16). The Cervical Cancer Screening Study carried out in the United States found that a BMI greater than 29 increases the risk of HPV infection and its progressing to CC (17, 18). In Mexico is high the prevalence of obesity in women (30 to 40%) and a study found that a high energy intake and obesity were observed in women with HPV (19).

Radiotherapy (RT) in pelvic area (period of 6 weeks in average) generates adverse effects such as diarrhea (15% at onset and a 84.7% at finish RT), vomiting (19% at onset and 65% a at finish RT), nausea (39% at onset and a 45% at finish RT) enteritis, colitis and proctitis cause intestinal malabsorption, enterocolitis, ulcers, stenosis and suboclusive symptoms (20). 83% of patients with RT in the pelvic area in the past lost weight during treatment (21–23). According to the evidence, the individualized nutritional treatment must be part of cancer treatment especially in CC that helps to reduce adverse effects of RT. The nutritional objectives must focus on reducing fat-free mass loss and maintaining its functionality as well as reducing the adverse effects generated by the toxicity of RT and improving prognosis cancer (24). Ravasco et al., evaluated the RT toxicity in patients with colon cancer in the abdominal-pelvic area found that 65% of these patients (who received only standard recommendations) had radiotherapy-induced toxicity 90 days after treatment while that the group who received an individualized nutritional intervention only 9% of the patients presented it (25).

Due the benefits of nutritional intervention on the maintenance of body composition (preserving fat free mass) in cancer during RT and its association with reducing adverse effects (mainly gastrointestinal) (26, 27), the aim of the present study was to evaluate the benefits on individualized nutritional therapy with counseling on decrease weight loss and gastrointestinal symptoms, compared with historical cohort group on adverse effects. We hope that these outcomes could be considered in pharmacology research, especially in repurposing drugs.

## Methods

### Study Population

Guanajuato is located in central Mexico, has 5,265,529 inhabitants (28), of which 51.7% are women (41% works at home). Guanajuato has a population of 657 513 emigrants (12.48% of the total population in the state) mainly to the United States. Migration is known to be a risk factor for increasing HPV exposure in women (29, 30). Since 2012 in Guanajuato has been implemented the program “Prevention and control of women’s cancer” for addressing caused of mortality of CC (31). Last epidemiologic analysis showed a rate of 4.7 death per 100,000 habitants in Guanajuato state in 2018 and 4.2 per 100,000 habitants in 2019 (32).

### Study Design

We conducted a longitudinal design with two comparation groups. The inclusion criteria were,for both groups, to have a medical diagnosis of CC with weekly radiotherapy in a public or private hospital, to have 18 years and over, to have been born in any city in the state of Guanajuato, medium to low socioeconomic and accept the informed consent. Participants who intake dietary supplements were not included and who did not have at least 80% weekly follow-up for cases or controls, were eliminated for the study. Non-probabilistic sample (consecutive cases by simple availability). A 100% of the cases and 70% of the controls (retrospective cohort) were selectec from the shelter “Jesús de Nazareth” located in Leon, Guanajuato.

### Study Groups and Recruitment Phase

The study groups were: 1) the intervention group (nutritional intervention and counseling -INC-) with 20 participants and 2) control group (retrospective cohort -CG-) with 9 participants.

**Figure 1:**
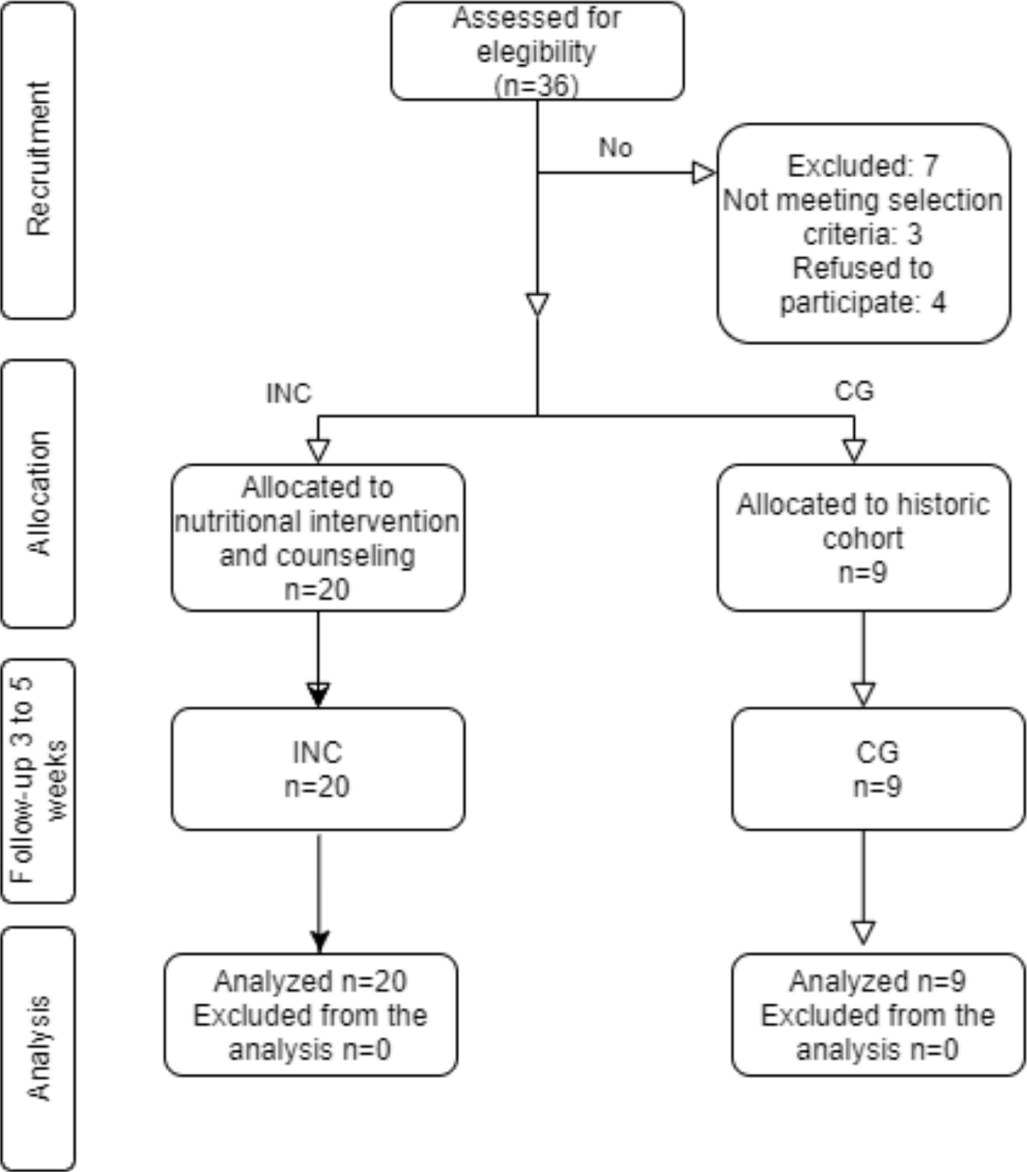
CONSORT flow diagram. For INC group, 36 patients were assesed for elegibility in the recruitment phase; 7 were excluded (three participants did not meet the inclusion criteria and four did not accept to participate). Nine participants of historical cohort were the control group. Twenty participants were allocated to nutritional intervention and conseling group. Both groups were followed up during RT of 3 to 5 weeks.

According to **Figure 1**, for INC group, 36 patients were assesed for elegibility in the recruitment phase; 7 were excluded (three participants did not meet the inclusion criteria and four did not accept to participate). Twenty participants were allocated to nutritional intervention and conseling group (INC). For retrospective cohort (CG) the sample size were nine. The historical cohort were study two years ago with the same characteristics. Both groups were followed up during RT of 3 to 5 weeks.

Intervention Group: Received a nutritional intervention (with individualized diet) and counseling during the radiotherapy treatment weekly (four to five weeks). The counseling was according to the gastrointesinal adverse effects and their tolerance. Before intervention, a complete nutritional evaluation was carried out body composition, dietary intake, adverse effects (gastrointestinal symptoms), glucose, hemoglobin, and blood pressure. The recommendations for energy and nutrient intake were based on ESPEN guidelines (33) for cancer patients. The dietary calculation was based with FAO/WHO/UNU, Harris-Bennedict formulas and ESPEN guidelines (suggest 25 to 30 kcal/kg/day with 1.2 to 1.5g protein/kg/day). Macronutrients were established of 20 to 30% for protein, 30 to 40% for lipids and 40 to 50% for carbohydrates and micronutrients were according to the *Recommended Daily Intake (RDI)* (34). The dietary recommendations were adjustment and individualized according to comorbidities presented in participants (diabetes, hypertension, hypothyroidism). The counseling was adjustmented according to the gastrointestinal adverse effects and food tolerance (for example, prescription of astringent diet when the diarrhea was presented or to increase energy density when anorexia appered), emphasizing the intake foods rich in carotenoids and antioxidants.

Control Group (Retrospective Cohort) (CG): *S*tandard counseling was prescribed, which included a list of foods allowed and to be avoided, as well as general recommendations for the control of adverse gastrointestinal symptoms. The CG data were draft from a study of 2016. The variables were the same for the INC group (body composition, nutritional status, adverse effects, and dietetic intake). The sample size were 9 participants. They received only standard counseling (recommendations) weekly throughout the RT.

### Nutritional Status

For the nutritional estatus, the anthropometric (body composition), dietary, biochemical, and clinical indicators were measured.

### Anthropometric Variable (Body Composition)

Body composition was measured with a bioimpedance analyzer (OMRON^®^ HBF-500INT). The definition for low fat mass was 9 to 23% percentage, acceptable value of 24 to 31% and unhealthy value for value ≥32%. Significant loss fat free mass was considered during radiotherapy treatment with ≥500 g. Body mass index (BMI) was considered malnutrition with <18.5 kg/m^2^, adequate value with 18.5 to 24.9 kg/m^2^, overweight with 25 to 29.9 kg/m2 and obesity grade 1 with 30 to 34.9 kg/m^2^ and obesity grade 2 with 35 to 39.9 kg/m^2^ (35). Arm, waist, and hip circumference (A value greater than 0.85 was considered cardiometabolic risk for Waist-to-hip ratio) was measured with a fiberglass tape (Vitamex^®^) according to the ISAK^®^ technique (36). All anthropometric measurements were performed by a previously standardized nutritionist.

### Diet

A 24-hour Recall was applied to assess the food and beverage intake (interview was in the last 24 h) with food replicas (NASCO^®^). The diet data was analyzed with Nutrikcal VO^®^ Software. The energy, macronutrients (carbohydrates, proteins, and lipids), and micronutrients (vitamins and minerals) were calculated for one day and once weekly. The adequacy percentage was calculated for energy, macronutrients and micronutrients intake respect. An aceptable value for adequacy percentage was considered with 90 to 110%.

$$Percentage\;of\;adequacy=\frac{\left(Actual\;consumption*100\right)}{Required\;consumption}$$

A consumption frequency questionnaire was applied with 8 food groups according to the Mexican System of Equivalent Foods (37). Vegetables, fruits, cereals and tubers, legumes, animal foods, dairy products, oils and fats, and sugars with the following frequencies: once a week, two to four times a week and daily.

### Biochemical Variables

Hemoglobin was measured from a capillary blood sample obtained with a sterile lancet with the Hemocue 201^®^ kit (specificity greater than 90% and a sensitivity of 80%) (38). Capilar blood glucose was measured with an Accu-Chek^®^ glucometer. The collection was carried out under postprandial conditions with a register of food intaked.

### Clinical: Adverse Effects

The main adverse effects of radiotherapy, mainly the gastrointestinal symptoms, were reported by the participants weekly, considering previous studies and the experience with retrospective cohort (CG) (20, 25).

*The VGS-GP* (Subjective global assessment-generated by the patient) was applied, the score was interpreted, A: good nutritional status; B: moderate malnutrition or risk of malnutrition and C: severe malnutrition. This instrument was applied at the beginning (first week of radiotherapy) and at the end of RT.

Blood pressure: Blood pressure was measured with a digital wrist baunometer (Omron^®^ R3), the participants were sitting and placing their wrist at heart level with the palm extended (39). This was measured at the beginning and at the end of the intervention.

Radiation Therapy Toxicity: The radiation toxicity was measured using the RTOG/EORTC acute toxicity scale for abdomen and pelvis (40). The acute toxicity scale was applied at the beginning (first week of radiotherapy) and at the end of radiotherapy.

Adherence to the intervention: Adherence to nutritional treatment was considered when the participants’ attendance is at 80% of sessions (3 weeks in average).

### Statistical Analysis

Descriptive analysis statistics were applied according to normal or non-normal distribution. For inferential analyzes, one-way Anova, Student’s t, Chi^2^ were applied. For the nonparametric variables, the Wilcoxon rank test, Student’s t test, and the Friedman test were used. To measure the effect of the intervention, the number needed to treat (NNT) was calculated. 80% power was considered with an alpha of *0.05*. The association of risk factors with the main variables (weight loss, fat-free mass and adverse effects) was calculated con Odds Ratio (95% confidence intervale). Statistical analyzes were performed with SPSS^®^ software V22 Free trial.

### Ethical Considerations

Participants received written informed consent with detailed explanation of the intervention. The research was carried out considering the Declaration of Helsinki, Nuremberg Code. The study was approved by the Bioethical Committee of the University of Guanajuato (No. CIBIUG-P22-2017).

## Results

**Figure 2:**
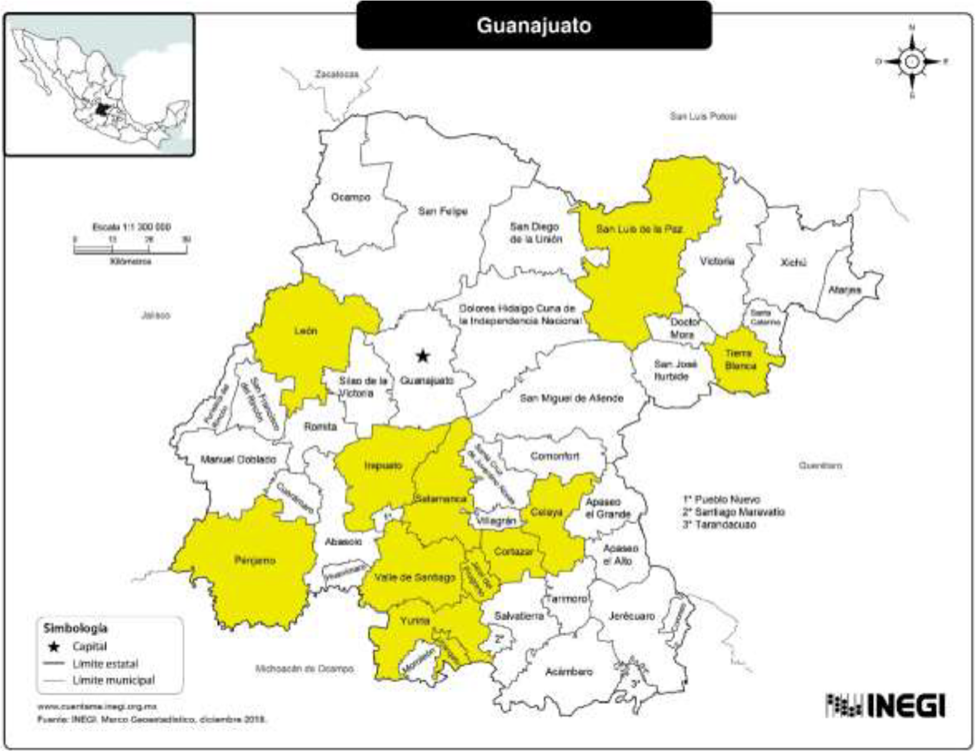
Cities origin from Guanajuato State of the participants of this study (Leon, Celaya, Irapuato, Salamanca, Cortazar, PEnjamo, San Luis de la Paz, Tierra Blanca, Jaral del Progreso, Valle de Santiago, Yuriria and Uriangato).

Their baseline characteristics showed that most participants were 51.5 years (rank 31 to 73 years) (p = 0.19). A 38.9% of participant were married for the INC group and 55.5% for the CG group (p = 0.18). A 50% of the participants in the INC group had primary complete. A 70% were housewives. The origin cities participants were originated are presented in **Figure 2**. A 44.5% of the participants in the INC group presented some comorbidities (diabetes mellitus, hypertension, hypothyroidism), while in the CG group only 22.2% presented some comorbidities (p = 0.259) (**Table 1**).

**Table 1:** Sociodemographic characteristics for both groups.

		INCn = 20	CGn = 9	P
	Age*	51.5(31–73)	51(35–83)	0.19*
Marital Status	Married	8(40)	5(55.5)	0.41**
	Single	5(25)	3(33.3)	
	Other	7(35)	1(11.1)	
Education level	Highschool	9(45)	–	
	Elementary	9(45)	–	
	None	2(10)	–	
Ocupation	Housewife	14(70)	4(44.4)	0.19**
	Employee	6(30)	5(55.5)	
Birthplace	Región del Bajío	13(65)	8(88.8)	0.18**
	Valles Abajeños	3(15)	1(11.1)	
	Sierra Gorda	4(20)	0(0)	
Comorbidity	Yes	9 (45)	2(22.2)	0.24**
	No	11 (55)	7(77.7)	
Stage of cancer	Stage I	2(10)	–	
	Stage II	12 (60)	–	
	Stage III	2(10)	3(33.3)	
	Stage IV	1(5)	–	
	No data	3(15)	6(66.6)	
Treatment	RT+CT	10(50)	3(33.3)	0.40**
	Previous Surgery	7(35)	2(22.2)	

INC, Nutritional Intervention and Counseling; CG, Control Group. RT+CT, Radiotherapy and chemotherapy. Data is expressed in percentages and frequencys. *Median (Rank). Mann Withney U test. **Fisher´s exact test.

### Anthropometric Variables (Body Composition)

**Figure 3:**
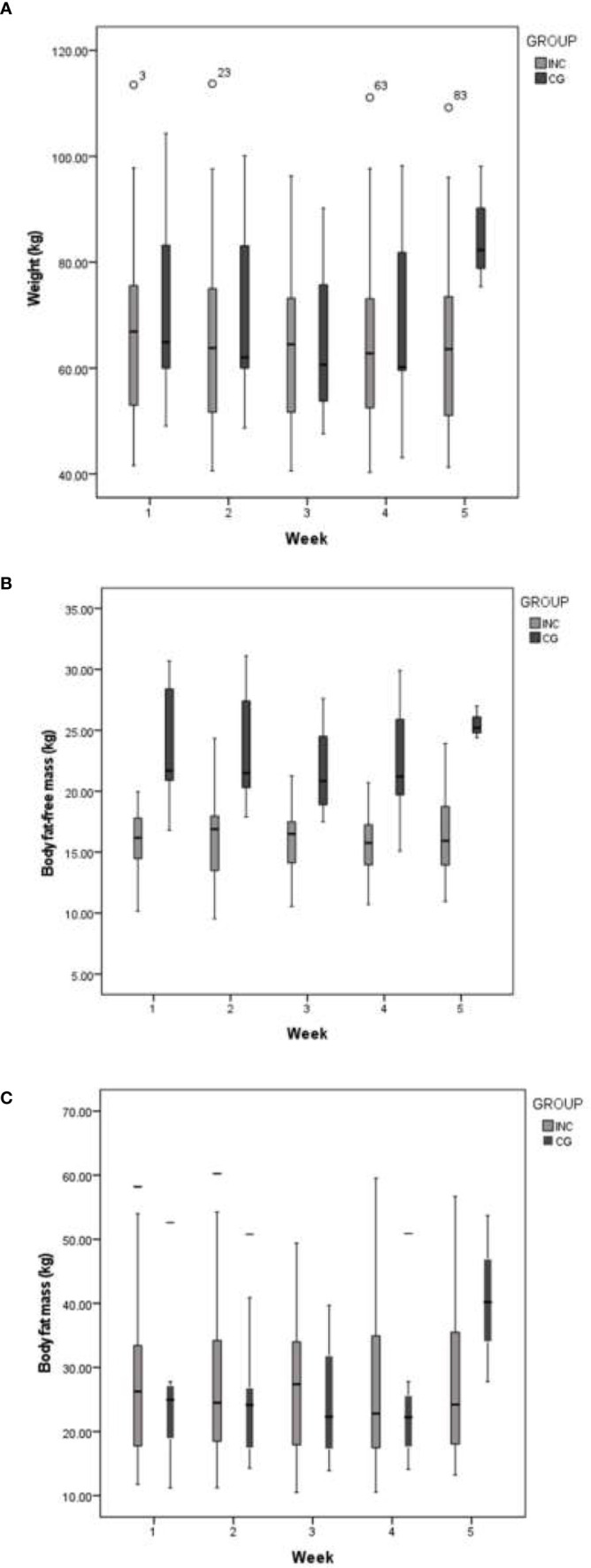
Weekly changes in weight, body fat-free mass and body fat mass in both groups during RT. **(A)** Body weight. **(B)** Body fat-free mass and **(C)** Fat mass.

Regarding anthropometric indicators, body weight and body mass index showed a significant decrease during the weeks for the INC group (*p = 0.013 and p = 0.043, respectively*). Likewise, there was a tendency to decrease fat-free mass and body fat observed mainly in the CG group. At the beginning of RT the INC group had overweight in 25%, 35% were obese and 40% an adequate nutritional status; in the CG group 44% were overweight, 22% were obese and 33% an adequate nutritional status. There were no changes in nutritional status in the INC group, but in the CG group one of the participants developed malnutrition at the end of the RT (**Table 2** and **Figure 3**).

**Table 2:** Anthropometric variables in Nutritional Intervention and Counseling group (NC) and Control Group (CG).

Indicator	Week 1		Week 2		Week 3		Week 4		Week 5		P value*	
	INCn = 20	CGn = 9	INCn = 19	CGn = 9	INCn = 17	CGn = 7	INCn = 17	CGn = 6	INCn = 15	CGn = 3	INC	CG
Weight (kg)	66.9 (42–114)	64.9 (49.1–104.3)	63.3 (41–114)	62 (48.7–100.1)	61.8 (41–96.3)	60.6 (47.6–90.2)	62.1 (40–111)	60.2 (43.1–98.2)	58.4 (41–109)	82.3** (75.4–98.1)	0.01	0.40
BMI (kg/m2)	27.7 (20–44)	26.9 (20.4–45.7)	27.6 (19–44)	26.8 (20.3–43.9)	26.8 (19–34.3)	25.9 (19.8–36.1)	26.6 (19–42.4)	26.1 (17.9–43.1)	25.7 (21–42.4)	28.3 (22.7–43)	0.04	0.40
Arm girth (cm)	28.4 (23–46.1)	30.3 (23–39.5)	28.7 (22–46)	29.5 (22.2–36.5)	29 (21–37.5)	27 (22.3–37.5)	28.5 (21–46)	28.3 (21.7–33.5)	28.5 (23–47.7)	31 (30–35.3)	0.47	0.15
Waist girth(cm)	89.3 (68–130)	88 (71–117)	88 (65–130)	84 (70–118)	88 (67–116)	82.5 (69.5–104)	85.5 (66–128)	89 (65.3–118)	85.5 (73–128)	91 (88.5–112)	0.18	0.06
Abdominal girth (cm)	96 (78–132)	98 (83–117)	96 (73–131)	95 (78–119)	95 (73–127)	92.2 (78–108)	94 (73–139)	94 (81.5–116)	97 (85–136)	99 (95.5–113)	0.66	0.25
Hips girth (cm)	102 (84–138)	106 (88–139)	100 (82–139)	97 (88.5–133)	100 (82–131)	96.5 (89–113)	99 79–138)	97 (85–135)	101 (85–139)	107 (104–130.5)	0.30	0.13
Fat free mass (kg)	16.7 (10–25.3)	21.7** (16.8–30.7)	16.9 (9.5–24.3)	21.5** (17.9–31.1)	16.5 (11–21.3)	20.8** (17.5–27.6)	16.2 (11–23.3)	21.2** (15.1–29.9)	15.9 (11–23.9)	25.8** (24.4–37)	0.57	0.40
Fat mass (kg)	29.1 (12–58.2)	25.5 (11.2–52.6)	28 (11–60.3)	25 (14.3–50.8)	27.4 (11–49.4)	22.3 (13.9–39.7)	22.8 (11–59.5)	23.4 (14.1–50.9)	24.2 (13–56.7)	46.9 (40.2–53.7)	0.30	0.40

*Friedman one-way repeated measure analysis of variance by ranks, **p < 0.05. Kruskall-Wallis test.

In the INC group the total weight loss during RT was of 1.1 kg (Rank 0.3 to 4.7 kg) while weekly weigh loss was 0.3 kg (rank 0.1 to 0.5 kg). For the CG group a statistically non-significant weigh loss of 2.7 kg (rank 0.9 to 6.2 kg) was observed throughout the five weeks while weekly was 0.9kg (rank 0.3 to 2kg). At comparing changes of weight weekly, mainly in the form of fat-free mass, for INC group the weight increased: last week (for weight *p = 0.044* and fat-free mass p= <0.001), week two (p <0.001), week three (p = 0.14), week four (p = 0.048) and week five (p = 0.008). A 55% of the participants in the INC group lost body fat with a median of 2,100g (range from 1100 to 2,700g) while in the CG group 73.6% lost body fat with a median of 1,070g (range from 370 to 4,100g) *(p= 1.000)*.

Weight loss in fat free mass (FFM) had a median of 410g (110 to 2,500g) in the INC group and 1,060g (100 to 2,500g) in the CG group; the weekly loss was 240g (150 to 460g) and 320kg (200 to 730g), respectively. A 37% of the INC group presented a loss greater than 500 g of FFM, while in the CG group 67% presented it (*RR = 0.55; 95% CI: 0.26 to 1.17*) with a reduction in the relative risk of 0.45 (95% CI= −0.17–0.74), an absolute risk reduction of 0.30 (95% CI= −0.08–0.67). The NNT was 4 (95% CI= 2 to −13).

### Diet

**Figure 4:**
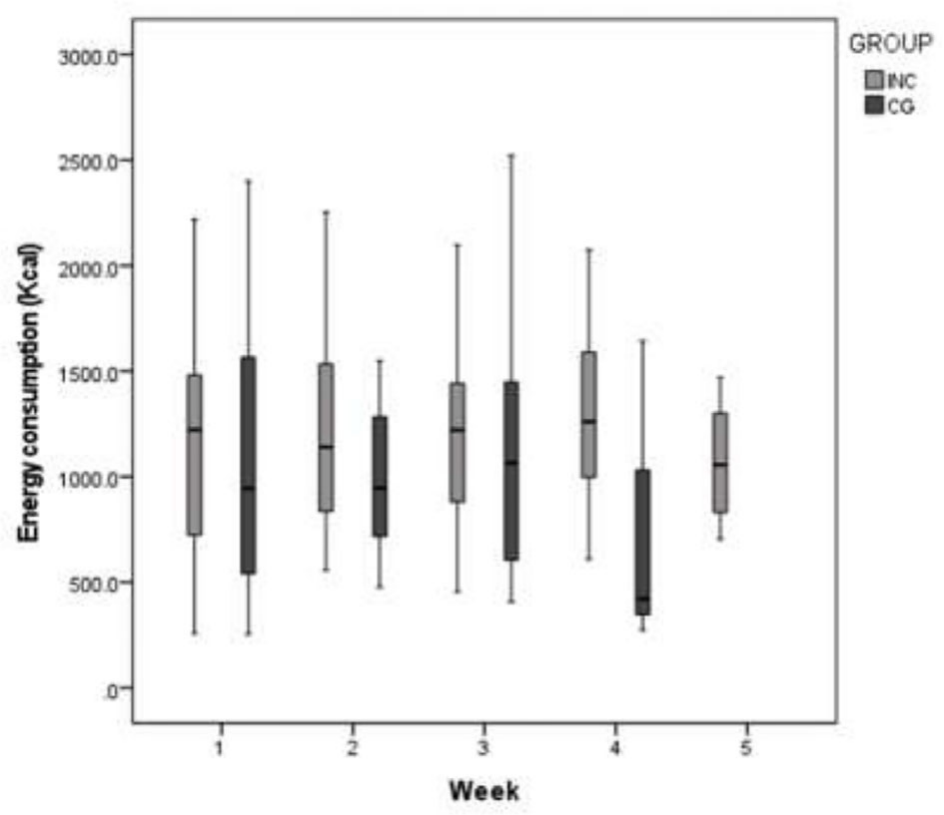
Weekly energy intake in both groups (INC, Nutritional intervention with counseling group; CG, historical cohort (control group).

Energy intake decreased weekly in both groups (**Table 3** and **Figure 4**). According to adequacy percentage in the INC group during RT the energy intake was in 60 to 80% except in week five with 65%. For the CG group, in the first week a 44% had an energy intake greater than 60%, in the third week was a 50% and in the fourth week a 33%. Respect to protein intake in first week for INC group a 50% of the participants had more than 60% of requirement while a 33% for CG group. For second, third and fourth week the protein, lipids and carbohydrates intake were higher in the INC group compared to the control CG. There were no data for the fifth week in the CG group. The rest of the nutriments are presented in **Tables 3**, **4**.

**Table 3:** CompaNutritional intake in comparison between groups.

	Week 1		Week 2		Week 3		Week 4		Week 5		P value*	
	INCn = 18	CGn = 9	INCn = 18	CGn = 9	INCn = 15	CGn = 8	INCn = 15	CGn = 3	INCn = 15	CGn = 0	INC	CG
Energy (kcal)	1222.5 (258–2219)	945 (255–2401)	1141 (557–2254)	947 (477–1550)	1222 (454–2097)	1065 (407–2552)	1260 (609–2076)	422 (273–1642)	1056 (704–1401)	–	0.50	0.81
Proteins (g)	50 (8.1–77)	39.2 (9.1–149)	39.9 (19.3–83.4)	20.1 (13.8–82.1)	45.5 (19.8–68.7)	31.15 (10.2–168)	42.6 (11.3–93.6)	31.4 (23–47.7)	38.8 (13.4–87.4)	–	0.58	0.89
Lípids (g)	35.3 (3.6–102)	26.12 (5.2–85.8)	37.5 (2.2–104)	26.40 (14.9–77)	25.9 (2.1–88.4)	21.73 (2.70–99.8)	27.4 (11.5–72.5)	5.57 (4.6–86–3)	25.7 (10.5–55.7)	–	0.30	0.80
Carbohidrates (g)	166 (29.5–354)	131.7 (59–259.8)	199.3 (108–350)	164.9 (51.6–278)	198.6 (96.9–335)	179.4 (62.4–238)	204 (111–377.8)	62.4 (34–192)	169 (96–294.9)	–	0.60	0.61
Sugar (g)	27 (6.9–97.5)	35.2 (11.1–135)	33 (9.7–150.2)	21.90 (3.8–73.6)	40.7 (8.9–111)	29.90 (4.2–81.5)	27.7 (3.4–78.2)	15.4 (0–24.4)	29.2 (4.5–65.9)	–	0.15	0.18
Fiber (g)	14.9 (3.8–27.3)	14.3 (5.6–31.3)	18.7 (4.6–27.9)	7.90** (5.3–21.6)	17.4 (4–40.8)	12.75 (2.2–24.2)	18.8 (5.8–56.4)	2.20 (1.6–17.6)	13 (4.9–47.6)	–	0.10	0.80
Vitamin A (µg RAE/day)***	1165 (132–9688)	355** (99–1097)	1444 (34–9409)	475 (24–2293)	1155 (18–8486)	709 (45–1512)	651 (214–10612)	66 (21–1024)	540 (88–6192)	–	0.24	0.89
Vitamin B12 (mg)	1.29 (0.19–12.2)	0.45 (0–8.3)	0.95 (0–5.13)	1.2 (0–3.97)	1.1 (0–10.9)	0.43 (0–6.4)	1.04 (0–12.7)	1.2 (0.41–1.3)	1.1 (0–4.23)	–	0.89	0.71
Vitamin C (mg)	37.2 (12–199.7)	40.1 (9.5–599)	56.1 (7–126.9)	43 (12.2–145)	41.7 (13.6–201.8}	42.4 (27.1–222)	39 (1.7–131)	40.8 (0.6–76.7)	32.4 (4.3–177)	–	0.49	0.53
Folic Acid (mcg)	109.5 (29–392)	96.6 (27.3–389)	92.3 (14–379)	54.2 (7.7–554)	148 (28–922)	75.7 (18.9–224)	140 (22–1160)	18.9** (5–87)	72 (15.3–833)	–	0.57	0.80
Iron (mg)	9.01 (1.34–14.4)	5.30 (2.4–15.2)	9.15 (1.03–19)	4.4** (2.6–14)	10.2 (2.1–18.9)	6.3 (1.7–20.6)	9.8 (3.9–29.4)	3.3 (1.7–12.8)	6.9 (1.24–24.9)	–	0.52	0.80
Selenium (mg)	25 (2–57)	18 (1–51)	18 (0–46)	11 (2–52)	16 (2–74)	17.5 (4–118)	19 (1–110)	21 (13–24)	6.5 (1–105)	–	0.12	0.68
Zinc (mg)	3.3 (0–7.9)	2 (0.4–10.6)	2.6 (0–8.6)	1.7 (0–9.5)	3.1 (0–6.7)	2.1 (0.3–9.5)	2.8 (2–9.5)	2.8 (1.4–6.1)	1.4 (0–6.4)	–	0.51	0.45

*Friedman one-way repeated measure analysis of variance by ranks, **p < 0.05. Kruskall-Wallis test.***RE: µg retinol estimated per day.

**Table 4:** Dietary intake and adequacy of energy and nutrients.

Intake adequacy percentage	Week 1		Week 2		Week 3		Week 4		Week 5		P value*	
	INCn = 18	CGn = 9	INCn = 18	CGn = 9	INCn = 15	CGn = 8	INCn = 15	CGn = 3	INCn = 15	CGn = 0	INC	CG
**Energy (Kcal)**	78 (15–147)	59 (14–150)	81 (33–125)	55 (25–103)	81 (27–137)	60 (25–148)	83 (37–153)	20 (18–102)	65 (45–113)	–	0.65	0.81
**Proteins g**	60 (9.8–102)	50 (10–187)	53 (23–95)	26 (14–102)	60 (24–92)	38 (12–197)	57 (14–138)	30 (29–59)	47 (19–134)	–	0.53	0.81
**Lípids g**	61 (7–198)	44 (8–160)	70 (4–178)	49 (24–154)	53 (4–189)	40 (5–176)	53 (21–155)	9 (7–161) **	52 (24–107)	–	0.41	0.89
**Carbohidrates g**	79 (14–202)	69.7 (26–143)	104 (59–158)	78 (25–139)	108 (46–148)	83 (33–119)	105 (57–223)	23 (18–96)	90 (54–163)	–	0.53	0.53

*Friedman one-way repeated measure analysis of variance by ranks, **p < 0.05. Mann-Whitney U test.

Lower intake for vitamin A was observed in the first week *(p = 0.04*); fiber (*p = 0.006*) and iron (*p = 0.03*); for the second and fouth week the carbohydrates (p = 0.04) and folic acid (p = 0.04) intake were lower for the CG group in comparison with INC.

### Biochemical Variables

There was a significant decrease in hemoglobin values, weekly in the INC group (*p = 0.009*); in second, third and fourh week the CG group had lower values (*p =0.016, p = 0.039*) (**Table 5**). At the end of treatment 21.3% of the INC group presented values less than 10 mg/dL. In the CG group, in the first week a 33% of the participants had hemoglobin values less than 10 mg/dL and at the end a 66%. maintaining blood sugar levels of 95–140 mg/dL. The glucose was maintained in normal values during RT.

**Table 5:** Weekly changes in blood pressure, glucose and hemoglobin.

	Week 1		Week 2		Week 3		Week 4		Week 5		P value*	
	INCn = 20	CGn = 9	INCn = 19	CGn = 9	INCn = 16	CGn = 9	INCn = 12	CGn = 6	INCn = 15	CGn = 3	INC	CG
Systolic blood pressure mmHg	113 (90–140)	110 (94–151)	110.5 (90–131)	110 (96–133)	108 (87–161)	112 (87–126)	106 (90–117)	111 (60–124)	107 (90–130)	118 (100–127)	0.224	0.102
Diastolic blood pressure mmHg	70 (60–89)	66 (53–87)	69 (58–81)	70 (56–83)	62 (57–97)	61.5 (52–81)	60 (52–70)	78 (54–80)	61 (50–80)	75 (64–80)	0.003	0.209
Capiilar blood glucose mg/dL	114 (70–219)	110 (93–417)	126 (72–167)	137 (97–317)	123 (90–306)	130 (106–161)	128 (80–169)	102 (89–216)	136 (74–290)	138 (117–298)	0.363	0.171
Hemoglobin g/dL	12. 2(10–15.3)	11** (6.9–15)	12.1 (9.6–14.6)	10** (6.7–15.7)	12.2 (8.6–14.1)	9.4** (6.3–12.3)	11.2 (8.7–15)	9.5 (8.9–13.6)	10.9 (8.3–13.6)	9.5 (9–12.4)	0.009	0.736

*Friedman one-way repeated measure analysis of variance by ranks, **p < 0.05. Kruskall-Wallis test.

### Clinical Indicators: Adverse Symptoms

**Figure 5:**
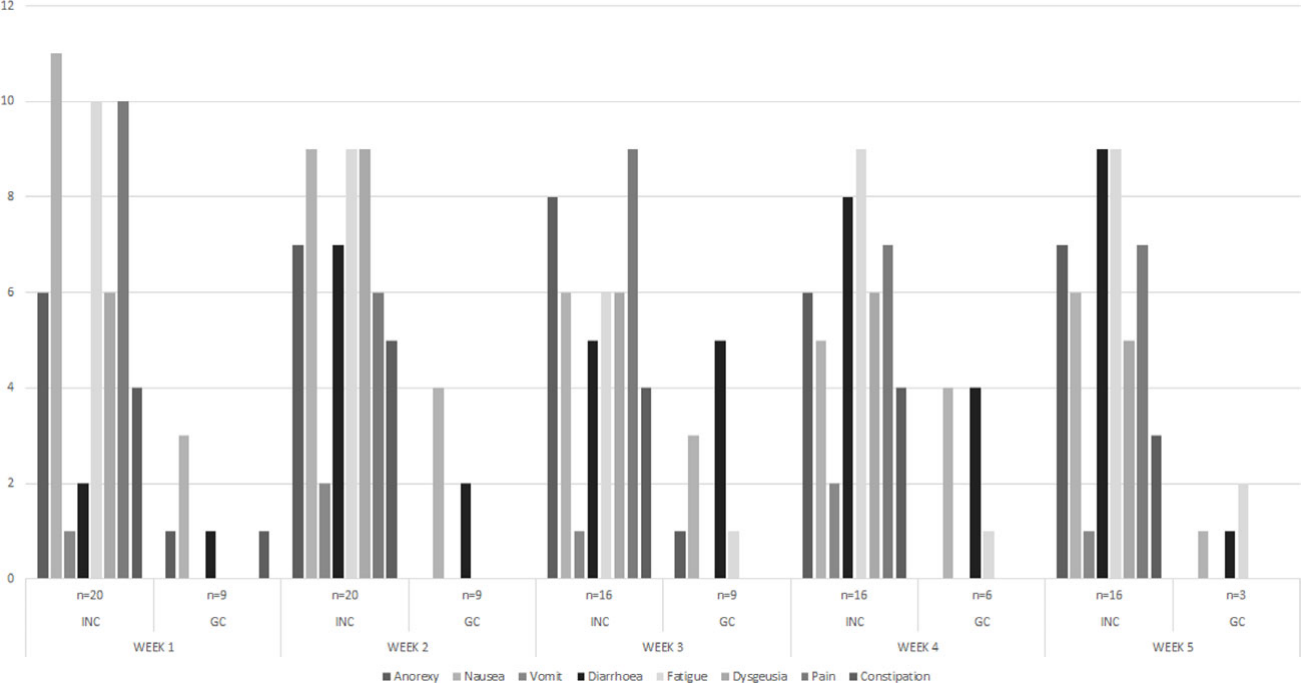
Weekly frequency adverse effects presented during RT in both group.

Regarding adverse symptoms for the first week, INC group had: nausea (58%), pain (53%), anorexia (32%) and dysgeusia (32%); in the last week, diarrhea (56%), fatigue (56%), anorexia (44%) and nausea (40%). For CG group the frequently adverse effect in the first week were nausea 33%, anorexia and a combination of diarrhea and constipation in one patient (**Table 6**). Dysgeusia was reported throughout the RT in the INC group and decreased it to 31% in the fifth week (**Figure 5**). 

**Table 6:** Weekly frequency of adverse effects during RT in both groups.

Adverse effect	Week 1			Week 2			Week 3			Week 4			Week 5		
	INCn = 20	CGn = 9	P value*	INCn = 20	CGn = 9	P value*	INCn = 16	CGn = 9	P value*	INCn = 16	CGn = 6	P value*	INCn = 16	CGn = 3	P value*
Anorexy	6(32)	1(11)	0.27	7(35)	0(0)	–	8(50)	1(11)	0.05	6(37)	0(0)	–	7(44)	0(0)	–
Nausea	11(58)	3(33)	0.28	9(45)	4(44)	0.97	6(37)	3(33)	0.83	5(31)	4(66)	0.13	6(40)	1(33)	0.89
Vomit	1(5)	0(0)	–	2(10)	0(0)	–	1(6)	0(0)	–	2(12.5)	0(0)	–	1(6)	0(0)	–
Diarrhoea	2(10)	1(11)	1.00	7(35)	2(22)	0.49	5(31)	5(55)	0.23	8(50)	4(66)	0.48	9(56)	1(33)	0.46
Fatigue	10(53)	0(0)	–	9(45)	0(0)	–	6(37)	1(11)	0.15	9(56)	1(16)	0.09	9(56)	2(66)	0.73
Dysgeusia	6(32)	0(0)	–	9(45)	0(0)	–	6(37)	0(0)	–	6(37)	0(0)	–	5(31)	0(0)	–
Pain	10(53)	–	–	6(30)	–	–	9(56)	–	–	7(44)	–	–	7(44)	–	–
Constipation	4(21)	1(11)	0.34	5(25)	0(0)	–	4(25)	0(0)	–	4(25)	0(0)	–	3(19)	0(0)	–

Data is expressed in frequencies and percentages. *Fisher´s exact test.

### Other Variables

Regarding blood pressure, a significant decrease in diastolic pressure was observed in the INC group throughout RT (*p = 0.003*). At the beginning of nutritional intervention, the INC group 85.7% had a low nutritional risk (12 participants) and 14.3% (2 women) had a moderate risk. At the end of RT (n = 11), 90% (10 participants) remained at low risk.

The radiation toxicity was measured only in INC group (n = 14) where only one participant had grade three and the rest remained in grade 1 at the end of RT.

### Association Analysis

An association was found between the presence of anorexia and a lower dietary intake in the first week (*p = 0.017*), but there was no association between this symptom and loss of body weight, fat mass or fat-free mass at the end of RT *(p= 0.082*). The presence of nausea, diarrhea, and dysgeusia were not associated with weight loss, fat mass, fat-free mass, and lower energy intake at the end RT *(p= 1.000*).

Nutrients and energy intake were not associated with weight loss, fat-free mass, and fat mass (p = 0.082). An intake less than 60% of the protein requirement was associated with a loss of fat-free mass greater than 500 g in the last week of radiotherapy in the INC group (p = 0.04), while an intake less than 60% for lipids requirement was associated with a loss of body weight greater than 500 g in the INC group in the last week of RT (*p= 0.028*).

A higher risk of had hemoglobin level less than 10mg/dL in the second week of RT in the CG group (*OR= 11.25; 95% CI= 1.57-80.3; p = 0.019). Other* risk factors for the CG group and INC as weight loss greater than 500 g and loss of fat-free mass greater than 500 g, energy intake less than 60%, serum hemoglobin at end of radiotherapy and the presence of symptoms such as anorexia are present in **Table 6**.

### Adherence to the Intervention

About 27 participants who were initially considered for the INC group, 55% of them attended the 100% of nutritional interventional while a 45% attended 4 sessions.

## Discussion

Based on the immunological aspect associated with diet, there is evidence that an individualized nutritional intervention can be effective to improve nutritional intake, conserve nutritional status and quality of life and with a reduction in radiation toxicity (41). In the present study, individualized nutritional intervention did not decrease the adverse symptoms compared with retrospective cohort, but to maintain the body weight, fat-free mass, fat mass and diastolic blood pressure in the participants. Likewise, a higher energy and nutrient intake was observed in the intervention group.

It is known that malnutrition due to low body mass index (<18.5kg/m^2^) and weight loss more than 5% are predictive indicators for developing radiation toxicity (42). In this study, 10% of INC group and 33% of CG group, presented loss weight greater than 5% after RT. It has been found that there is a positive association between body mass index and cervical cancer (*HR 1.10; CI 99%, 1.03–1.17*) (43). Furthermore, women whit overweight or obesity used not attend screening for CC (44). A 60% and 66% of the participants in both groups in this study had obesity or overweight, respectively. The obesity is known to promote inflammation through immune system dysfunction (45). Obesity is also associated with low functional level and a greater number of comorbidities in cancer patients (46). It is substancial to adrees both overweight and obesity as an especial issue that should be discussed and considered in the individualized nutrition therapy in cancer patients. The relationship between adipose tissue, muscle mass and other tissues in the body composition of the cancer patient has important clinical implications. Bioimpedance analysis is accessible tecnique, portable and inexpensive method that give important data on body composition. Although few studies have analyzed the body composition of patients with cervical cancer, an association has been found between low values ​​of phase angle with postoperative complications and hospital stay (47–49).

A study in 2004, carried out in patients with head and neck cancer, showed that an early and individualized nutritional intervention can decrease fat-free mass loss and considered such loss clinically significant when it was greater than 500 g, during radiotherapy, since this involves to an impact on physical functionality (50). In our study, body composition analysis demonstrated a trend toward greater fat-free mass loss in the CG group in comparison with INC group. The intention-to-treat analysis allowed to consider that receiving an individualized nutritional intervention could be a protective factor for a fat-free mass loss greater than 500g, which was 42% less likely that this occurs in the group with INC and that four is the number of patients that must be treated with an INC to avoid losing more than 500g in fat-free mass.

Respect to the dietary intake, it decreased during all treatment and weekly. A tendency to be lower was observed in the participants of the CG group, although it was not statistically significant. Interestingly, it has been observed in various studies that nutritional intervention can improve and increase dietary intake (20, 51).

In the aspect of dietary prescription to cancer patients, it is known that nutritional support could increase the speed of tumor growth however, when nutritional status is compromised, complications may be greater and have an impact on survival prognosis (52). Current recommendations encourage compliance with the energy requirement that covers from 20 to 30 kcal/kg/day, when an individualized calculation is not available, intake less of than 60% (individual requirement) is considered deficient (53), in this study the 65% of the participants in the INC group had a consumption greater than 60% and increased and maintained it at 75% the following weeks, compared to the control group, where initially 44% covered in the first week, in the third week a 50% and in the fourth week only 33% of the participants cover the requeriments.

The protein intake was in tendency to be higher (although not statistically significant) in the INC group compare with CG group. In both groups it was less than recommended in the intervention that was carried out individually, according to the needs of the participant (a contribution of 1 to 1.3 g/kg is recommended). This, together with the deficient consumption of micronutrients, which is also caused by the low dietary consumption, could constitute a significant risk to maintain an adequate weight and, therefore, avoid the problems caused by possible malnutrition. Even so, the consumption of folic acid, vitamin A and iron was significantly higher in the INC group, in the first, second and fourth weeks, respectively. This decrease in the consumption of micronutrients has also been found in other studies (54); however, it is still necessary to study the supplementation of some vitamins in these patients, so the recommendation is to follow the daily intake recommended by the national academy of sciences and for critically ill patients, evaluating each case individually (54, 55).

It is important to mention that adherence to nutritional treatment is an aspect that has a great impact on the results derived from the interventions; for example, one study showed that the risk of developing colorectal cancer can decrease up to 30% by having adequate adherence to the nutritional recommendations of the World Cancer Research Fund and the American Institute for Cancer Research (55). Multiple factors influence the adherence to nutritional treatment of patients; One of the main limitations for the participants to have an adequate fulfillment of their nutritional needs has been the attention to general recommendations that are given to them when they start their treatment. These recommendations generally restrict food that they consume daily and to which they have access. For example, the consumption of legumes (mainly beans and lentils) is maintained at least once a week in approximately 60% of the participants, despite the restriction.

Food availability and its relationship with the presence of cancer in a specific population, in 2018 an ecological study was conducted where found a correlation between with red meat intake, calories and animal fat with colorectal cancer (*r = 0.59 and r2 of 0.29; r = 0.56 and r^2^ of 0.16; r = 6*, respectively) while a weak correlation was found with the availability of fruits and vegetables (56). These results should be analyzed, since it has not been considered individually, for example, individual high availability would not necessarily indicate a high consumption of food. Several factors influence this situation in the cancer patient for example gastrointestinal tolerance, sociodemographic variables. The anemia is an important variable that must be considered as a predictor factor in the prognosis of patients with chemotherapy and radiotherapy, mainly with values less than 10mg/dL in the last two weeks of treatment (57, 58). Although glucose during treatment had not significant increases because its measurement was collected in post-prandial condition; even so, the maximum ranges present an increase higher than expected in a healthy person. A factor that may have had an influence was the presence of participants with comorbidities such as diabetes, as well as the association between elevated serum glucose levels with recurrence and mortality in patients who do not have diabetes (59). In the present study, only 25% of the patients in INC group, at the end, presented <10 mg/dL, compared to the CG group with values greater than 60%.

In the aspect of adverse effects, when comparing with a study carried out under the same methodology with the scale (RTOG/EORTC) with which the INC group was evaluated, in our study, in patients with chemo-radiotherapy, the frequency of participants with nausea, anemia and vomiting, it was lower (40, 50, and 6%, respectively) than that of the sample evaluated by said study (73.3, 69.2, and 20.9%). Frecuently diarrhea (56% in the INC group vs. 51.6%) was found by Izmajłowicz et al. Regarding lifestyle, risk factors such as smoking, should be addressed with re-relevance, since smoking has a RR of 2.4 (95% CI: 1.7, 3.4) and the risk remains even with smoking cessation (*RR = 1.6, 95% CI: 1.0–2.7*) (60). The risk increases in women who smoked for a period of 16 years (OR: 3.23, 95% CI: 1.33, 7.69), and continue in recurrent smokers who consume more than 20 cigarettes a day (*OR: 2.57, 95% CI: 1.49 to 4.45*) (61).

It is important to mention that a limitation with comparison groups was the methodology to obtain the frequency of adverse effects that it was different in the CG group. In this sense, further research, and comparison of groups in which the variable of adverse effects has been measured with the same method is suggested. However, the outcomes in INC group could be used in pharmacological studies, with synergy of therapies and improve the prognosis of women with cervical cancer.

According to these findings, the implications and favorable effects of supervision, professional accompaniment in a nutritional intervention can be identified, which pays a methodology to be integrated for the research study of drug reuse and the nutritional intervention itself. The evidence shows an important synergy between some dietary components and drugs for the treatment of diseases associated with both lifestyle (hyperlipidemias, diabetes), and some, whose risk factors may be more complex, such as cancer (62, 63). A study by Kindelwal et al, in 2018, for example, showed that selenium-induced toxicity could be effective in treating breast cancer by considering an immunotherapeutic approach that can reduce the debilitating side effects that are associated with breast cancer drugs (64). The search for treatments that generate inhibition of cell proliferation mechanisms in Cancer, such as the inhibition of the ubiquitin system, proteasome, which is responsible for the degradation of proteins in the cell, in 80 to 90% is increasingly attractive through the reuse of drugs. In this sense, there is a growing interest in the use of some natural compounds such as flavonoids, polyphenols, isoflavones, curcumin and other compounds that are found intrinsically in food, the use of which could act in synergy with the anticancer drug, with a potential lower toxicity (65).

There are several factors that increase the cancer patient’s susceptibility to malnutrition, this negatively impacts the prognosis, progression, and decrease in response to treatment (**Table 7**). Oncological treatments such as chemotherapy, radiotherapy, chemo-radiotherapy, or surgery can compromise food intake, nutrient absorption and affect the patient’s nutritional status (66). INC is an adjunct to the treatment of various disorders, however, the evidence regarding cervical cancer is limited. The effect of various drugs already known on various mechanisms that can improve or complement the effect of basic therapies has been analyzed. For example, evidence shows that drugs such as emetine, fluorosalan, sunitinib malate, bithionol, narasin, tribromsalan, lestaurtinih can inhibit NF-kappaB (NFKB1) signaling, by inhibiting the phosphorylation of IkappaBelpha (NFKBIA), a transcription factor that plays an important role in the growth of cells in CC (67). On the other hand, the role of zoledronic acid as a drug that could inhibit the proliferation of cervical cancer cell lines has also been studied, and in addition, in combination with paclitaxel or deoxorubin, it showed better inhibition of Ras oncogenes (68). the proposed mechanisms also include immunomodulation through PD-1/PD-L1 blockade, which has shown a response in up to 13 to 17% of gynecological cancers, probably due to the immunosuppressive effect that occurs in the microenvironment in tumors. gynecological and altered vasculature. It has been observed that the effect of this mechanism can improve benefits in conjunction with radiotherapy (69).

**Table 7:** Main risk factors associated with the nutritional variables in both groups.

Nutritional variables	INCn = 20	CGn = 9	OR (CI95%)	P value*
Weight loss greater than 500 gr.	15	7	1.167 (0.180–7.564)	0.631
Fat free mass greater than 500 gr.*	9	2	1.778 (0.134–23.52)	0.579
Serum hemoglobin <10mg/dl (second week)	2	5	11.25 (1.57–80.3)	0.019
Serum hemoglobin <10mlg/dl (final week)*	2	2	15.00 (0.896–251.06)	0.088
Energy consumption less than 60%	5	4	2.240 (0.424–11.837)	0.407
Anorexia	14	1	0.333 (0.33–3.335)	0.633

*Fisher´s exact test.

The search for new drugs has improve the quality life and saved lives although they are expensive and require many years of research and the effectiveness of these depend on pharmacokinetics and pharmacodynamics variables of each drug. The efficacy of a drug can be compromised by deterioration gastrointestinal absorption and therefore nutritional deterioration. The repurposing of drugs consists of finding new therapeutic indications for existing drugs, and therefore reducing the research times involved in the study of drugs with the advantage of knowing their risks already studied (70, 71). For cervical cancer, the treatment is chemotherapy and radiotherapy, but both have adverse effects towards the maintenance of nutritional status, which increases morbidity and mortality and therefore the prognosis of the disease. The nutritional intervention plus nutritional counseling should be reporpused as an essential part of the clinical trials for drug validation as it may improve benefits for patients with CC (71).

The design of drugs takes several years and very high costs, for which counting on the reuse of drugs and therapies as a nutritional intervention and counseling raises great hopes, since the effects of treatments (in this case, local radiation) affect all cells. The scheme for CC is to proceed with chemotherapy and radiotherapy, two systemic treatments with known adverse effects and high toxicity. On the other hand, the drugs that are developed to treat cancer and other diseases require high costs, due to the need to analyze the aspects of pharmacodynamics (absorption, distribution, elimination) where aspects such as nutritional status, gastrointestinal absorption and hemodynamic status stable. They are key to measure the effectiveness of a treatment, which is why this study supports a methodological proposal (72, 73).

It is urgently necessary to develop drugs and more effective, economic strategies that seek to decrease the resistance that has been generated to current drugs (some patients develop resistance to chemotherapy) or increase sensitivity to existing drugs or repurposing drugs.

Recommendations: The energy and nutritional intake was maintained with the intervention with adequate adherence to the intervention with, was better and remained constant weekly in the participants who received the intervention, although it remained below what was recommended. Food security could be an important factor to meet the requirements in this population; therefore, individualized nutritional intervention should consider this aspect, in the sense of food availability. Furthermore, it is recommended to conduct clinical trials with a greater sample size.

What this study provides: The effect of a nutritional intervention and individualized counseling vs. standard counseling from a historical cohort on adverse effects and body composition, and the findings are expected to contribute to the methodology in the study of cancer drug repurposing and improving the effectiveness of these.

Our study had several limitations. First, the sample size for the retrospective cohort group. Second, use the retrospective cohort as the control group. Third, the equipment used to measure blood pressure, the American Heart Association recommends using a home blood pressure monitor that measures upper arm blood pressure and not using wrist or finger blood pressure monitors; Fourth measure only one a week for food intake. On the other hand, many of the findings are consistent with other studies, but the retrospective cohort will observe differences despite these limitations. Nutritional treatment is known to help prevent nutritional deterioration and improve prognosis during radiotherapy and chemotherapy, but weekly monitoring is required, at least as in our study. This is important for drug studies, where these variables must be controlled in order to measure the effect of different drugs.

## Conclusion

An individualized nutritional intervention and counseling with weekly monitoring and supervision throughout radiotherapy treatment demonstrated an impact on the maintenance of muscle mass, weight, hemoglobin, and a dietary intake above 70% of the recommendations for dietary intake and, a decrease in gastrointestinal adverse effects. Overweight and obesity found should be considered as part of the treatment for nutritional intervention and controlled counseling. These first findings reinforce the benefits of an individualized nutritional intervention to be implemented and reporpused in studies of drug reuse, achieving maintenance of nutritional status and a decrease in adverse effects, mainly anorexia and nausea as well as anemia. Improving the efficacy of pharmacological treatments and therefore improving their quality of life.

## Data Availability Statement

The raw data supporting the conclusions of this article will be made available by the authors, without undue reservation.

## Ethics Statement

The studies involving human participants were reviewed and approved by Comité Institucional de Bioética en la Investigación de la Universidad de Guanajuato (CIBIUG). The patients/participants provided their written informed consent to participate in this study.

## Author Contributions

All authors contributed to the article and approved the submitted version.

## Conflict of Interest

The authors declare that the research was conducted in the absence of any commercial or financial relationships that could be construed as a potential conflict of interest.
